# Mechanism of Action of Decitabine in the Treatment of Acute Myeloid Leukemia by Regulating LINC00599

**DOI:** 10.1155/2023/2951519

**Published:** 2023-02-22

**Authors:** Fan Du, Ting Jin, Li Wang

**Affiliations:** ^1^Department of Gastroenterology, Union Hospital, Tongji Medical College, Huazhong University of Science and Technology, Wuhan, China; ^2^Department of Hematology, The Central Hospital of Wuhan, Tongji Medical College, Huazhong University of Science and Technology, Wuhan, China

## Abstract

**Objective:**

Acute myeloid leukemia (AML) is a heterogeneous malignancy with a low long-term survival rate. The aim of this study was to investigate the effects of decitabine (DAC) treatment cell proliferation and apoptosis in AML and role of the expression of LINC00599 and, consequently, miR-135a-5p.

**Materials and Methods:**

Human promyelocytic leukemia cells (HL-60) and human acute lymphatic leukemia (CCRF-CEM) cells were treated with various concentrations of DAC. Cell proliferation in each group was detected using the cell counting kit 8. For each group, apoptosis and reactive oxygen species (ROS) levels were detected using flow cytometry. Reverse transcription polymerase chain reaction (RT-PCR) was performed to examine the expression of lncRNA LINC00599. The expression of apoptosis-related proteins was analyzed using western blotting. The regulatory relationship between miR-135a-5p and LINC00599 was verified by constructing miR-135a-5p mimics, miR-135a-5p inhibit, wild type LINC00599 3′-untranslated region (UTR), and mutant LINC00599 3′-UTR. Ki-67 expression in the tumor tissues of nude mice was detected using immunofluorescent assays.

**Results:**

Both DAC and LINC00599 Inhibit groups were able to significantly reduce the proliferation of HL60 and CCRF-CEM cells, increase apoptosis, upregulate the expression of Bad, cleaved caspase-3, and miR-135a-5p, downregulate the expression of Bcl-2, and elevate ROS levels in cells, with these effects being more pronounced after combined treatment with DAC and LINC00599 Inhibit. In comparison to mimic NC, the miR-135a-5p mimic group significantly decreased the relative fluorescence activity ratio of LINC00599 3′-UTR wild-type CCRF-CEM cells. The LINC00599 Inhibit and miR-135a-5p mimic groups exhibited substantially reduced proliferation of HL60 and CCRF-CEM cells, increased apoptosis, upregulated Bad, cleaved caspase-3, and miR-135a-5p expression, along with downregulated Bcl-2 and LINC00599 expression and increased ROS levels in cells; these effects were more pronounced after LINC00599 Inhibit was combined with miR-135a-5p mimics. In vivo experiments revealed that both DAC and LINC00599 Inhibit were able to considerably reduce the long diameter, short meridian, volume, and mass of tumors, increase miR-135a-5p expression, and decrease LINC00599 and ki-67 expression in tumor tissues of nude mice. This effect was more pronounced when the DAC and LINC00599 Inhibit were used in combination.

**Conclusion:**

DAC regulates the expression of miR-135a-5p by regulating the expression of LINC00599, which in turn affects cell proliferation, apoptosis, and tumor proliferation. Our findings provide a theoretical basis for improving the clinical outcome of AML.

## 1. Introduction

Acute myeloid leukemia (AML) is a bone marrow disease that is caused by a dysfunction in hematopoietic stem cells due to genetic alterations in the precursor cells, resulting in the overproduction of tumorigenic clonal myeloid stem cells [1]. Despite recent advances in the strategies to alleviate AML, long-term survival rates remain low due to the development of drug resistance and high relapse rates following currently available chemotherapies [2, 3]. As a result, new therapeutic targets to improve the clinical outcomes of patients with AML are urgently required.

Decitabine (DAC), also known as 5-aza-2'-deoxycytidine nucleoside, is a natural adenosine analog of 2′-deoxycytidine acid. DAC is the most potent specific inhibitor of DNA methylation and inhibits DNA methyltransferase and reduces DNA methylation, impeding tumor cell proliferation as well as preventing the development of drug resistance. DAC is widely used as a drug for the treatment of leukemia, but studies pertaining to the mechanism of action of DAC in the treatment of leukemia cells are scarce [4, 5]. Non-coding RNAs (ncRNAs) are functional small RNA molecules that do not translate into proteins, opening new perspectives for the diagnosis, prognosis, and treatment of AML [2, 6]. MicroRNAs (miRNAs), circular RNAs (circRNAs), and long non-coding RNAs (lncRNAs) are involved in the transcription and translation of genes that function as regulatory ncRNAs. LncRNAs are ncRNAs longer than 200 nucleotides [7]. The lncRNAs are classified as intergenic, intronic, sense, and antisense [8]. With the development of RNA-sequencing technology, an increasing number of ncRNAs have been found to be associated with the development of AML; these ncRNAs play a key role in the proliferation, differentiation, and apoptosis of leukemic cells and have the potential to serve as prognostic biomarkers [2, 9]. DAC regulates lncRNA expression, while DAC treatment in cell lines with hypermethylation caused dose- and time-dependent lncRNA expression and demethylation [10]. MiRNAs are involved in AML chemoresistance and transporter-mediated drug resistance through apoptosis, cell cycle, and adenosine triphosphate (ATP) binding in various ways [11, 12]. Recent studies have revealed that aberrant expression of lncRNAs in AML can alter the function of specific miRNAs, thereby promoting the initiation, maintenance, and progression of leukemogenesis [13, 14].

Therefore, in this study, LINC00599-disrupted cells were treated with DAC to evaluate apoptosis in leukemic cells and to determine whether leukemia can be treated using DAC by affecting LINC00599 expression, which in turn impacts miR-135a-5p expression, influencing cell proliferation and apoptosis. In addition, we verified if DAC affects tumor proliferation through LINC00599, using a tumorigenesis assay in nude mice.

## 2. Materials and Methods

### 2.1. Cell Lines, Reagents, and Other Materials

Human promyelocytic leukemia cells (HL-60) and acute lymphatic leukemia cells (CCRF-CEM) were obtained from the Shanghai Cell Bank of the Chinese Academy of Sciences. DAC (A119533) was purchased from Aladdin (China). Lipofectamine® 2000 (11668-027) was purchased from Invitrogen (China). Opti-MEM (M5650) was purchased from Sigma (USA). The dual luciferase reporter gene assay kit (RG027) was purchased from Beyotime (China). The cell counting kit 8 (CCK8) (C1706) was purchased from Bioswamp (China). The SYBR FAST qPCR Master Mix (KM4101) was purchased from KAPA Biosystems (USA). The oligo DT18/miR-135a-5p RT Primer (3806), recombinant RNase inhibitor (2313A), and PrimeScript II RTase (2690A) were purchased from TAKARA (Japan). BCA protein concentration assay kit (PC0020) was purchased from Solarbio (China), DAB (DA1010) was purchased from Solarbio (China), and all antibodies (anti-Bad PAB32756; anti-Bcl-2 PAB30599; anti-cleaved-caspase-3 MAB37300; anti-GAPDH PAB36269; goat anti-rabbit IgG SAB43714) were purchased from Bioswamp (China).

### 2.2. Cell Culture and Drug Therapy

The frozen HL-60 and CCRF-CEM cells were placed from the liquid nitrogen tank into a water bath set at 37°C, and after they were completely thawed, the cell suspension was pipetted into a centrifuge tube. In the tube, 4 mL of complete medium (basic medium along with serum, antibiotics, and other substances) was added, and the whole solution was centrifuged at 400 × *g* for 3 minutes, then resuspended in 1 mL of medium, transferred to a culture flask. Complete medium (serum, antibiotics, and other substances to the basic medium, 4 mL) was added to the flask, and the culture was incubated at 37°C in a 5% CO_2_ incubator. The cells in culture were treated with different concentrations of DAC (0 *μ*M, 0.01 *μ*M, 0.1 *μ*M, 1 *μ*M, and 2 *μ*M) for 24 hours, 48 hours, and 72 hours. The optimal duration of activity and DAC concentration were determined using CCK8, quantitative reverse transcription polymerase chain reaction (qRT-PCR), and flow cytometry.

### 2.3. Construction of Vectors

By synthesizing lncRNA LINC00599-shRNA1, lncRNA LINC00599-shRNA2, and lncRNA LINC00599-shRNA3 target genes, the vector (pLKO.1-EGFP) was double digested and ligated with the target genes at 16°C overnight. The wild type (wt)-LINC00599 3′-untranslated region (UTR) and mutant-LINC00599 3′-UTR genes were constructed, the vector (pmirGLO) was digested, and the target gene was ligated to the vector using ligase at 16°C overnight. The DNA fragments to be transformed were added to tubes containing TOP10 receptor cells and placed on ice for 30 minutes. The cells were then placed in circulating water at 42°C, heat-stimulated for 90 seconds, transferred to an ice bath, allowed to cool for 1–2 minutes, and 200 *μ*L of super-optimal broth with catabolite repression (SOC) liquid medium was added. The medium was warmed to 37°C in a water bath, and the tubes were transferred to a shaker which was set at 37°C and incubated at 220 rpm for 45 minutes to allow the cells to recover and express the plasmid-encoded resistance marker genes. The transformed receptor cells were transferred to a Luria–Bertani agar medium containing the corresponding antibiotics, and the plates were inverted and incubated at 37°C. After 12–16 hours of incubation, a number of monoclonal shake bacteria were randomly selected and sequenced to verify the positive clones.

### 2.4. Transfection of Vectors

The constructed miR-135a-5p mimic/inhibitor vector was transfected into CCRF-CEM cells. The constructed wt-LINC00599 3′-UTR and MT-LINC00599 3′-UTR vectors were transfected into CCRF-CEM cells. LINC00599-shRNA1, LINC00599-shRNA2, and LINC00599-shRNA3 were transfected with CCRF-CEM and HL-60 cells. The following were the transfection steps: 5 × 10^5^ cells were resuspended in 1.5 mL of complete medium prior to transfection, 100 pmol miRNA was diluted in 250 *μ*L of Opti-MEM followed by dilution of 5 *μ*L of Lipofectamine® RNAiMAX in 250 *μ*L of Opti-MEM, and the final solution was allowed to stand at room temperature for 5 minutes. The cells were then mixed and incubated at room temperature for 20 minutes. The complex (500 *μ*L) was added to the wells of the plate containing the cells along with 1.5 mL of fresh medium. The plate was gently shaken back and forth, and the plate was placed in a 5% CO_2_ incubator at 37°C. Following 4 hours of transfection, the medium was replaced with fresh medium, and the cells were incubated for 24 hours. The expression of the transferred genes, miR-135a-5p and LINC00599, was detected using qRT-PCR. Dual luciferase activity was detected using the dual luciferase reporter gene assay kit. Some methods of this experiment refer to previous research [15].

### 2.5. CCK8 Assay to Assess Cell Proliferation

The cells were divided into control, DAC, LINC00599 Inhibit, and LINC00599 Inhibit + DAC groups. The proliferation rate of cells in each group was determined using the CCK8 assay. HL-60 and CCRF-CEM cells were treated for 48 hours according to the experimental groups: control group, LINC00599 Inhibit, miR-135a-5p mimic group, miR-135a-5p Inhibit group, LINC00599 Inhibit + miR-135a-5p mimic group, and LINC00599 Inhibit + miR-135a-5p Inhibit group, and the proliferation rate of cells in each group was detected. CCK8 test procedure: cells from each group were collected, the concentration of the cell suspension was adjusted, and the cells were divided into 96-well plates (3 × 10^3^ cells/well, 100 *μ*L per well) and incubated overnight at 37°C in a 5% CO_2_ incubator. Following various group treatments, the cells were cultured for 48 hours. Each well of the cell culture plate was then treated with 10 *μ*L of CCK8 solution, and the culture was further continued for 4 hours. Finally, the absorbance value of each well was measured at 450 nm.

### 2.6. qRT-PCR Analysis

In a homogenization tube, 1 × 10^6^ cells of CCRF-CEM and HL-60 cells and 1 mL of TRIzol were mixed. The mixture was homogenized with a homogenizer for 20 seconds and then immediately placed on ice for total RNA extraction and reverse transcription. The SYBR Green PCR kit (KM4101; KAPA Biosystems) was used for qRT-PCR amplification. The reaction procedure is as follows: initial denaturation at 95°C for 3 minutes, denaturation at 95°C for 5 seconds, denaturation at 95°C for 5 seconds, annealing at 56°C for 10 seconds, extension at 72°C for 25 seconds (40 cycles), extension at 65°C for 5 seconds, and extension at 95°C for 50 seconds. Data were analyzed using the 2^-*ΔΔ*Ct^ method. The primer sequences used were as follows: miR-135a-5p–F: GGGGTATGGCTTTTTATTCCT, miR-135a-5p–R: AACTGGTGTCGTGGAGTCGGC; LINCOO599-F: AGGAAGTCGTTGGGCTATGT, LINCOO599-R: CTACAGGGAGGGCGTGAG; U6-F: CTCGCTTCGGCAGCACA, U6-R: AACGCTTCACGAATTTGCGT; GAPDH-F: CCTTCCGTGTTCCTAC, GAPDH-R: GACAACCTGGTCCTCA.

### 2.7. Western Blotting

Total protein was extracted from the cells, and the protein concentration in each group was determined using a BCA kit. Protein (20 *μ*g) was added to the gels (formulated with 12% isolate and 5% concentrate), with 80 V for 40 minutes for concentrate and 120 V for 50 minutes for isolate, and 90 V for 50 minutes at constant pressure for membrane rotation, with 5% skim milk powder sealed at room temperature overnight at 4°C. Primary antibodies (anti-Bad 1 : 1000, anti-Bcl-2 1 : 1000, anti-cleaved-caspase-3 1 : 1000, anti-GAPDH 1 : 1000) were added and the mixture was incubated at room temperature for 1 hour. Following membrane washing, the secondary antibody goat anti-rabbit IgG 1 : 10000 was added and the mixture was incubated for 1 hour at room temperature. The electrochemiluminescence (ECL) luminescent solution was added to a fully automated chemiluminescence analyzer, and the grayscale values of the relevant bands were read using TANON GIS software. Three replicates were performed for each group.

### 2.8. Flow Cytometry

#### 2.8.1. Apoptosis Detection

Cells (1 × 10^6^) were taken, centrifuged at 4°C for 5 minutes, and the supernatant was discarded. Phosphate-buffered saline (PBS) was added to the pellet, centrifuged at 4°C for 5 minutes followed by resuspension in 200 *μ*L PBS; 10 *μ*L Annexin V-FITC and 10 *μ*L PI were added and incubated at 4°C for 30 minutes followed by the addition of 300 *μ*L PBS and flow detection and analysis using NovoExpress analysis software.

Reactive oxygen species (ROS) detection: 2′,7′-dichlorofluorescein diacetate (DCFH-DA) was diluted with serum free culture solution up to a final concentration 10 *μ*mol/L. The cell concentration was adjusted to 1 × 10^6^/mL. The mixture was incubated in 5% CO_2_ at 37°C in a cell culture incubator for 20 minutes. The cells of each group were then collected, resuspended in 500 *μ*L PBS (1 × 10^6^/mL), and ROS were detected using flow cytometry. The analysis was performed using the NovoCyte analysis software.

### 2.9. In Vivo Studies

A total of 24 BALB/c nude mice (6-week-old) were used. All animals were purchased from Changzhou Cavins Laboratory Animals Co. Following seven days of acclimatization, 5 × 10^6^ CCRF-CEM cells were inoculated subcutaneously into the right axilla to initiate tumor formation. After the nude mice became tumor-bearing, the animals were divided into four groups: control: the same dose of saline was gavaged once a day for 14 days; DAC group: 1 mg/kg DAC was gavaged once daily for 14 days; LINC00599 Inhibit group: LINC00599 interfering vector (20 *μ*g/animal) was injected *in situ* at the site of tumor formation twice a week for 2 weeks; and LINC00599 Inhibit group + DAC: 1 mg/kg DAC was gavaged once daily along with injecting LINC00599 interference vector (20 *μ*g/animal) at the site of tumor formation twice a week for 2 weeks.

### 2.10. Immunohistochemistry

The tumor tissues were dewaxed and sectioned (3 *μ*m), and the dewaxed sections were soaked in 100% alcohol I, 100% alcohol II, 95% alcohol, 85% alcohol, and 75% alcohol for 5 minutes. The tissues were then rinsed with tap water for 10 minutes, repaired using 1 mM Tris-EDTA buffer solution at high pressure (125°C, 103 kPa) for 18 minutes, naturally cooled, and washed with PBS. Primary antibody (anti-ki-67 1:200) was added, incubated overnight at 4°C followed by the addition of secondary antibody (MaxVision TM HRP-Polymer anti-mouse/rabbit IHC Kit). Antibody-added sections were incubated at 37°C for 60 minutes, stained with DAB, re-stained with hematoxylin for 3 minutes, dehydrated, and photographed with a microscope. The Leica Application Suite graphics system was used for acquiring the relevant parts of the sample. Hematoxylin stained the nuclei blue, and DAB showed a brownish-yellow color.

### 2.11. Statistical Analysis

SPSS 22.0 software was used for statistical analysis. Data were represented as the mean ± standard deviation (SD) of three (in vitro) or six (in vivo) independent replicates. A one-way analysis of variance (ANOVA) was used to compare multiple groups, and the least significant difference (LSD) test was used for a two-way comparison of means. Differences with *P* < 0.05 were considered statistically significant.

## 3. Results

### 3.1. Selection of Optimal Concentration and Time of Action of DAC

**Figure 1:**
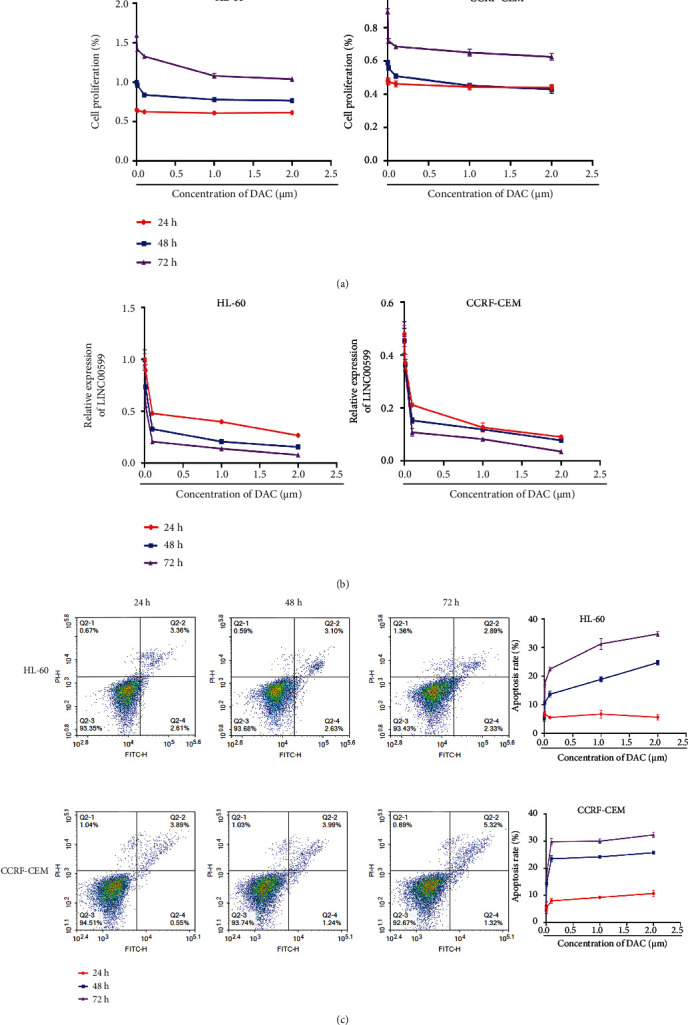
Selection of the optimal concentration and DAC activity duration. (a) Detection of cell proliferation in each group using CCK8. (b) qPCR detection of expression of lncRNA LINC00599. (c) Flow cytometric detection of apoptosis.

HL60 and CCRF-CEM cells were treated with different concentrations of DAC for 24 hours, 48 hours, and 72 hours. The proliferation of cells in each group was detected using the CCK8, and the results showed (Figure 1(a)) that the proliferation of cells did not change significantly at 24 hours of DAC treatment, while when treated with DAC for 48 hours and 72 hours, the value-added of cells decreased sequentially with the increase in DAC concentration, and the value-added of the cells treated with DAC for 48 hours was always lower than that at 72 hours. The expression of the lncRNA LINC00599 was detected using qPCR (Figure 1(b)), and the relative expression of LINC00599 in cells showed a sequential decrease with increasing concentrations of DAC at different time points. Apoptosis was detected using flow cytometry (Figure 1(c)), and no significant change was observed in the apoptosis of cells at 24 hours of DAC treatment, whereas the apoptosis of cells increased sequentially with the increase in DAC at 48 hours and 72 hours of treatment. As a result, based on these findings, we selected the concentration of 2 *μ*M DAC for follow-up experiments.

### 3.2. DAC Mediates the Expression of LINC00599 to Promote Apoptosis

**Figure 2:**
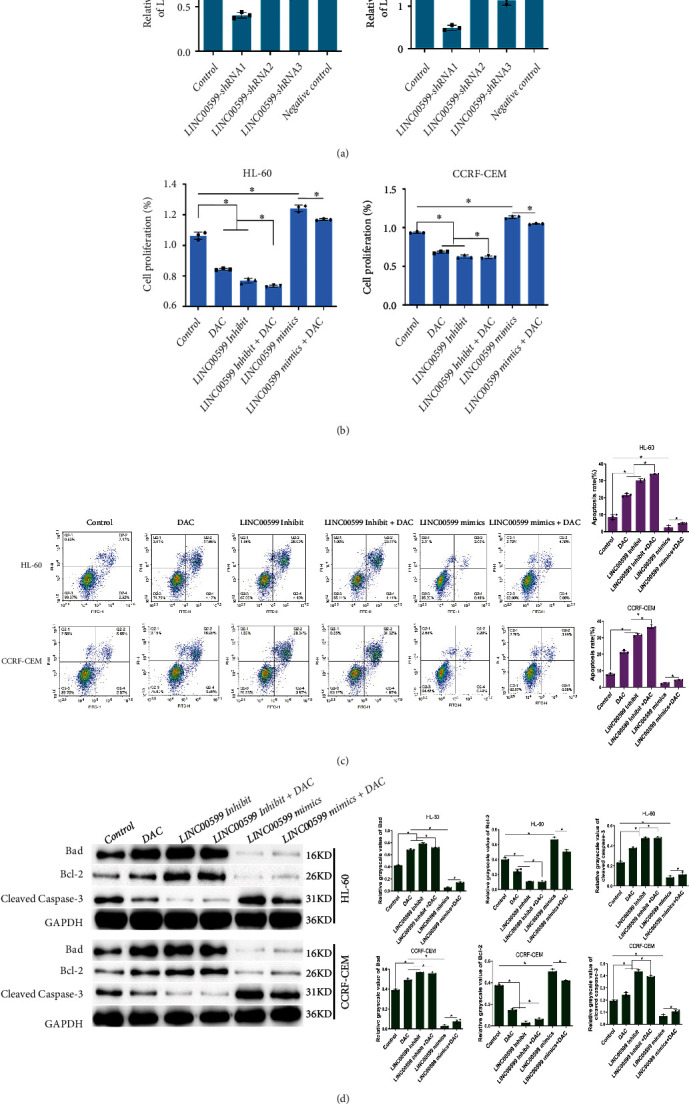
DAC mediated lncRNA expression for apoptosis induction. (a) Results of qPCR analysis examining interference efficiency. (b) Results of CCK8 assay to detect cell proliferation of each group of cells after DAC mediated lncRNA expression. (c) Flow cytometric analysis for the apoptosis of each group of cells. (d) Western blotting results detecting the expression of apoptosis-related proteins in each group of cells.

We constructed three different interference plasmids: LINC00599-shRNA1, LINC00599-shRNA2, and LINC00599-shRNA3. The interference efficiency was examined using qPCR (Figure 2(a)), and LINC00599-shRNA1, LINC00599-shRNA2, and LINC00599-shRNA3 were all able to significantly reduce the relative expression of LINC00599 in both HL60 and CCRF-CEM cells compared with the control group (*P* < 0.05), with LINC00599-shRNA1 demonstrating the best inhibitory effect. As a result, we selected LINC00599-shRNA1 for the inhibition of LINC00599 expression. The proliferation of cells in each group after DAC-mediated lncRNA expression was detected using the CCK8 assay (Figure 2(b)). Compared to the control group, the proliferation of HL60 and CCRF-CEM cells in the DAC group and the LINC00599 Inhibit group decreased significantly (*P* < 0.05), whereas the proliferation of cells in the LINC00599 mimic group increased significantly. The proliferation of HL60 and CCRF-CAM cells was significantly lower in the DAC + LINC00599 Inhibit group than that in the DAC and LINC00599 Inhibit groups (*P* < 0.05). According to the apoptosis rate detected using flow cytometry in each group (Figure 2(c)), the apoptosis rates of HL60 and CCRF-CEM cells in the DAC group and the LINC00599 Inhibit group increased significantly (*P* < 0.05), whereas that of the LINC00599 mimic group decreased significantly compared to the control group. The apoptosis rates of HL60 and CCRF-CEM cells were significantly higher in the DAC + LINC00599 Inhibit group than in the DAC and LINC00599 Inhibit groups (*P* < 0.05). We detected changes in apoptosis-related proteins using western blotting (Figure 2(d)). Compared to the control group, the expression of Bad and cleaved caspase-3 in HL60 and CCRF-CEM cells in the DAC group and LINC00599 Inhibit group was significantly upregulated (*P* < 0.05), while the expression of Bcl-2 was significantly downregulated (*P* < 0.05). Bad and cleaved caspase-3 expression was significantly downregulated, while Bcl-2 expression was significantly upregulated in the LINC00599 mimic group. Additionally, Bad and cleaved caspase-3 expression was significantly upregulated (*P* < 0.05), while Bcl-2 expression was significantly downregulated in HL60 and CCRF-CEM cells in the DAC and LINC00599 Inhibit groups compared with the control group (*P* < 0.05). Furthermore, Bad and cleaved caspase-3 expression was significantly upregulated (*P* < 0.05), while Bcl-2 expression was significantly downregulated (*P* < 0.05) in HL60 and CCRF-CEM cells in the DAC + LINC00599 Inhibit group compared with the DAC and LINC00599 Inhibit groups. These findings indicate that both DAC treatment and the inhibition of LINC00599 expression can promote apoptosis in HL60 and CCRF-CEM cells, and that the inhibition of LINC00599 combined with DAC was the most effective in promoting apoptosis.

### 3.3. Effect of DAC Mediates LINC00599 on miR-135a-5p and ROS

**Figure 3:**
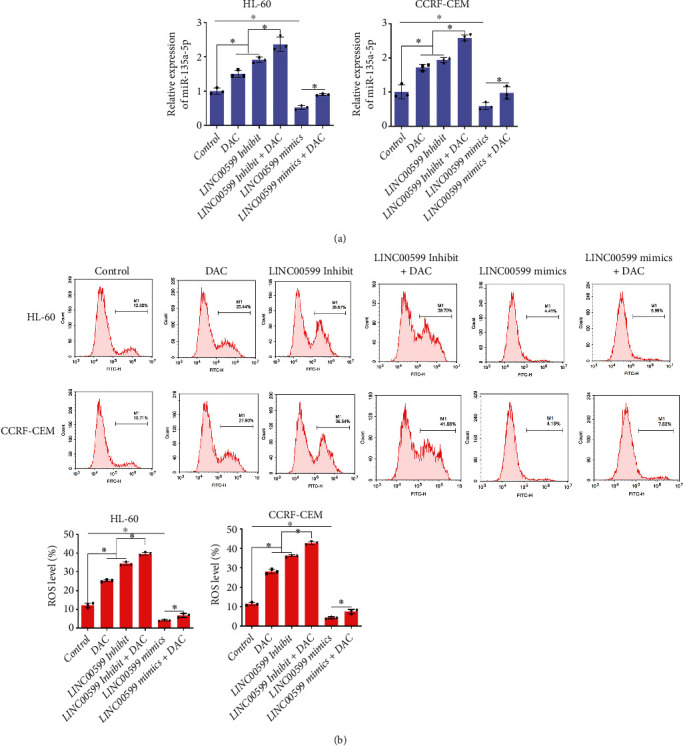
The influence of DAC on the effect of lncRNA on miR-135a-5p and reactive oxygen species (ROS) levels. (a) qPCR detecting miR-135a-5p expression in cells and (b) results of flow cytometry analysis detecting ROS levels in each group of cells.

The expression of miR-135a-5p was detected in the cells using qPCR (Figure 3(a)). Compared to the control group, the expression of miR-135a-5p in HL-60 and CCRF-CEM cells in the LINC00599 Inhibit group was significantly upregulated (*P* < 0.05), whereas the expression of miR-135a-5p in the LINC00599 mimic group was significantly downregulated. The expression of miR-135a-5p was significantly upregulated in HL60 and CCRF-CEM cells in the LINC00599 Inhibit + DAC group than in the DAC group (*P* < 0.05). The ROS levels in the cells of each group were detected using flow cytometry (Figure 3(b)). Compared to the control group, ROS levels in HL60 and CCRF-CEM cells in the DAC group and LINC00599 Inhibit groups were significantly higher (*P* < 0.05), whereas ROS levels in the LINC00599 mimic group were considerably lower. ROS levels in HL-60 and CCRF-CEM cells were significantly higher in the DAC + LINC00599 Inhibit group than in the DAC and LINC00599 Inhibit groups (*P* < 0.05). These results suggested that DAC-mediated lncRNA expression promotes apoptosis in HL-60 and CCRF-CEM cells by regulating miR-135a-5p expression and ROS levels.

### 3.4. Effect of LINC00599 Binding to miR-135a-5p on Apoptosis

**Figure 4:**
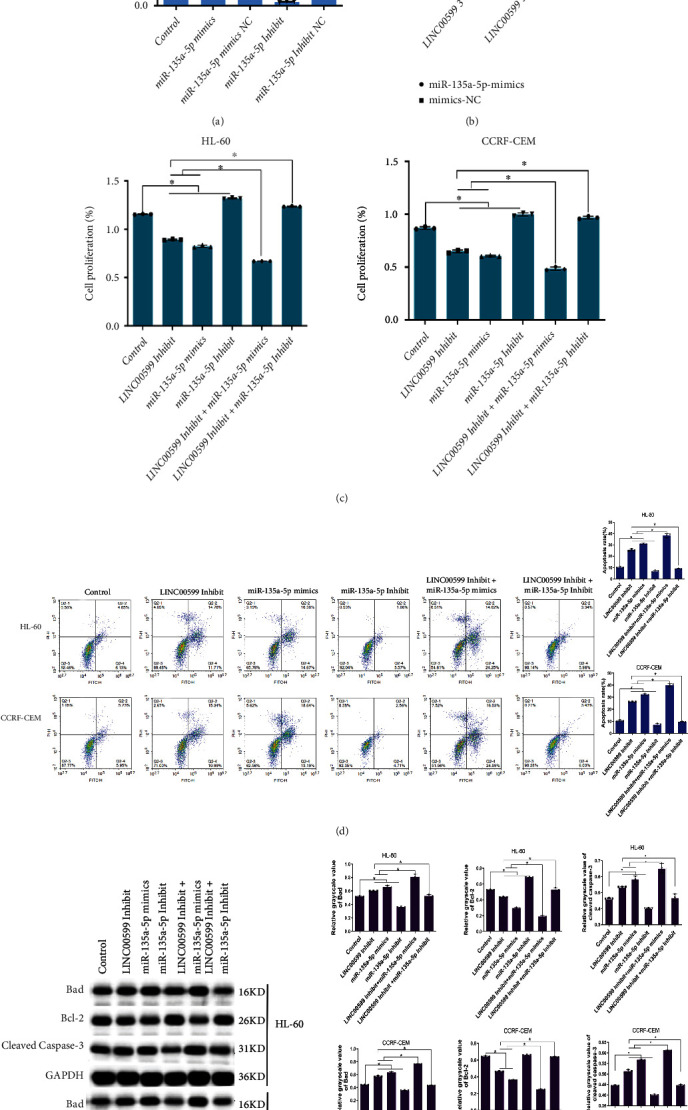
Effect of LINC00599-miR-135a-5p interaction on apoptosis. (a) qPCR determining vector efficiency. (b) Transfection efficiency evaluation by detecting luciferase activity. (c) CCK8 assay revealing the proliferation of each group of cells. (d) Flow cytometric analysis for detecting apoptosis in each group of cells. (e) Western blotting analysis for the detection of apoptosis-related proteins in each group of cells.

Overexpression and interference vectors of miR-135a-5p were constructed and transfected into CCRF-CEM cells, and vector efficiency was determined using qPCR (Figure 4(a)). The relative expression of miR-135a-5p was significantly upregulated in the cells of the miR-135a-5p mimic group compared to the miR-135a-5p NC group (*P* < 0.05), and the relative expression of miR-135a-5p in the cells of the miR-135a-5p Inhibit group was significantly downregulated compared to that in the miR-135a-5p Inhibit NC group (*P* < 0.05). The dual luciferase plasmid empty vector, wt LINC00599 3′-UTR wild type and mutant LINC00599 3′-UTR mutant were constructed and transfected into CCRF-CEM cells, and the efficiency of transfection was verified by detecting the luciferase activity (Figure 4(b)). The miR-135a-5p mimic group was able to significantly reduce the relative fluorescence activity ratio of LINC00599 3′-UTR wild-type CCRF-CEM cells compared with mimics NC (*P* < 0.05). The proliferative capacity of the cells in each group was detected using CCK8 (Figure 4(c)), and compared with the control, the LINC00599 Inhibit group and miR-135a-5p mimic group were able to significantly decrease the proliferation of HL60 and CCRF-CEM cells (*P* < 0.05), and miR-135a-5p Inhibit group significantly increased the proliferation of HL60 and CCRF-CEM cells (*P* < 0.05); the LINC00599 Inhibit + miR-135a-5p mimic group was able to significantly reduce the proliferation of HL60 and CCRF-CEM cells compared with the LINC00599 Inhibit and miR-135a-5p mimic groups (*P* < 0.05). The apoptosis of each cell group was detected using flow cytometry (Figure 4(d)); when compared to the control, the LINC00599 Inhibit and miR-135a-5p mimic groups significantly increased apoptosis (*P* < 0.05), while the miR-135a-5p Inhibit group significantly decreased apoptosis (*P* < 0.05); the LINC00599 Inhibit + miR-135a-5p mimic group significantly increased apoptosis of HL60 and CCRF-CEM cells compared with the LINC00599 Inhibit and miR-135a-5p mimic groups (*P* < 0.05). We detected the expression of apoptosis-related proteins using western blotting (Figure 4(e)), and compared with the control, the LINC00599 Inhibit and miR-135a-5p mimic groups were able to significantly decrease Bcl-2 expression (*P* < 0.05) and increase Bad and cleaved caspase-3 expression (*P* < 0.05) in HL60 and CCRF-CEM cells; miR-135a-5p Inhibit group significantly increased the expression of Bcl-2 in cells (*P* < 0.05) and decreased the expression of Bad and cleaved caspase-3 in cells (*P* < 0.05). Compared with the LINC00599 Inhibit and miR-135a-5p mimic groups, the LINC00599 Inhibit + miR-135a-5p mimic group showed significantly decreased Bcl-2 expression in HL60 and CCRF-CEM cells (*P* < 0.05) and increased Bad and cleaved caspase-3 expression (*P* < 0.05). This result indicated that the inhibition of LINC00599-binding miR-135a-5p overexpression promoted apoptosis in HL60 and CCRF-CEM cells.

### 3.5. Effect of LINC00599 Combined with miR-135a-5p on Cellular ROS

**Figure 5:**
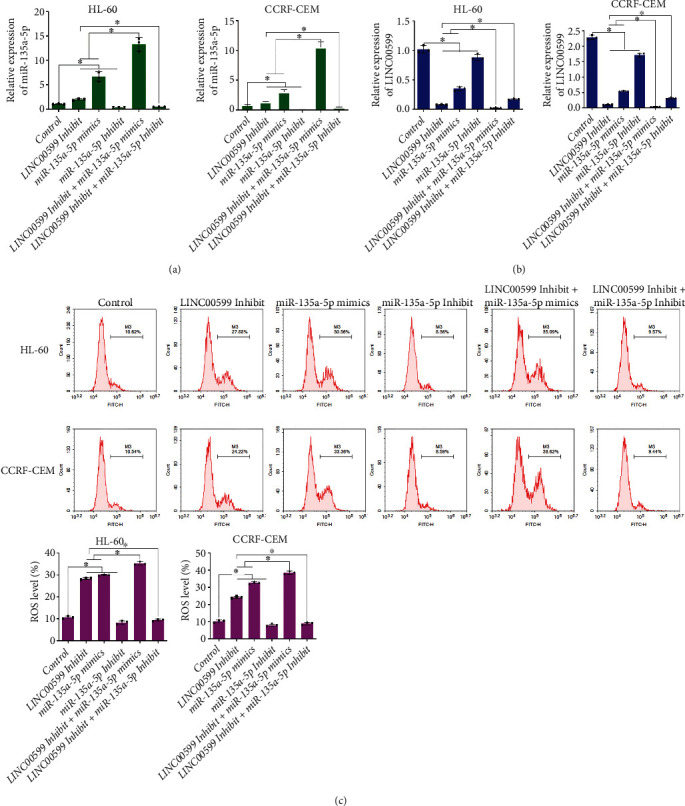
Effect of LINC00599 binding to miR-135a-5p on cellular reactive oxygen species (ROS). (a) qPCR analysis detecting the expression of (a) miR-135a-5p and (b) LINC00599 in cells. (c) Flow cytometric analysis detecting the level of ROS in each group of cells.

Expression of LINC00599 and miR-135a-5p in cells was detected using qPCR (Figures 5(a) and 5(b)), and compared with the control group, the LINC00599 Inhibit and miR-135a-5p mimic groups significantly increased the expression of miR-135a-5p in HL60 and CCRF-CEM cells (*P* < 0.05) and decreased the expression of LINC00599 in cells (*P* < 0.05), and the miR-135a-5p Inhibit group significantly reduced the expression of miR-135a-5p in cells (*P* < 0.05). Compared with the LINC00599 Inhibit group and miR-135a-5p mimic group, the LINC00599 Inhibit + miR-135a-5p mimic group significantly increased expression of miR-135a-5p (*P* < 0.05) and decreased expression of LINC00599 (*P* < 0.05). The level of ROS in the cells was detected using flow cytometry (Figure 5(c)), the LINC00599 Inhibit group and miR-135a-5p mimic group were able to significantly increase ROS levels in HL60 and CCRF-CEM cells compared to the control group (*P* < 0.05). The LINC00599 Inhibit + miR-135a-5p mimic group significantly increased the level of ROS in cells compared to the LINC00599 Inhibit group and miR-135a-5p mimic group (*P* < 0.05). These results suggest that LINC00599 binding to miR-135a-5p promotes apoptosis in HL60 and CCRF-CEM cells by increasing ROS levels.

### 3.6. DAC-Regulated LINC00599 Affects Tumor Proliferation in Nude Mice

**Figure 6:**
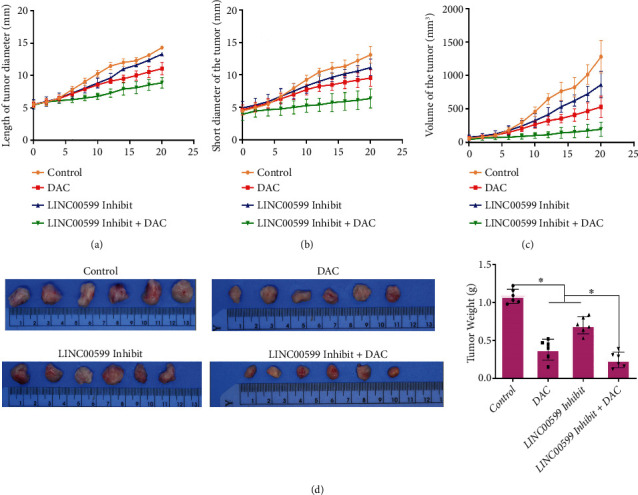
DAC affecting tumor proliferation in nude mice via LINC00599. Calculation of the tumor (a) long diameter, (b) short diameter, (c) volume, and (d) mass.

The CCRF-CEM cells were injected subcutaneously into nude mice. The long diameter (Figure 6(a)), short meridian (Figure 6(b)), and volume (Figure 6(c)) of the tumors in the nude mice revealed that as the treatment time of DAC, LINC00599 Inhibit, and DAC + LINC00599 Inhibit increased, a trend was observed in that the long diameter, short meridian, and volume of tumors in nude mice decreased, and the combination of DAC + LINC00599 Inhibit had the best effect. After two weeks of treatment, sampling was performed, and the tumors were removed for weighing. The results are shown in Figure 6(d). Compared with the control group, both the DAC and LINC00599 Inhibit groups significantly reduced the mass of tumors in nude mice (*P* < 0.05). The DAC + LINC00599 Inhibit group significantly reduced tumor mass compared to the DAC and LINC00599 Inhibit groups (*P* < 0.05).

### 3.7. DAC-Mediated LINC00599 Affects the Expression of LINC00599, miR-135a-5p, and ki-67 in Tumor Tissues

**Figure 7:**
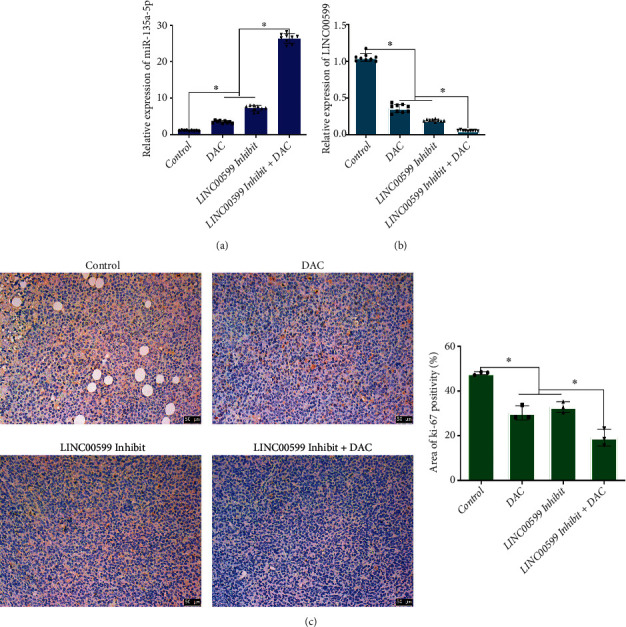
DAC influencing the expression of LINC00599, miR-135a-5p, and ki-67 in tumor tissues through LINC00599. qPCR analysis detecting the expression level of (a) miR-135a-5p and (b) LINC00599 in tumor tissues of each group. (c) Immunohistochemical analysis detecting ki-67 expression in tumor tissues.

The expression of LINC00599 and miR-135a-5p in each group of tumor tissues was detected using qPCR (Figures 7(a) and 7(b)). Both the DAC and LINC00599 Inhibit groups significantly increased the expression of miR-135a-5p (*P* < 0.05) and decreased the expression of LINC00599 (*P* < 0.05) compared with the control group. The DAC group + LINC00599 Inhibit group increased the expression of miR-135a-5p (*P* < 0.05) and decreased the expression of LINC00599 (*P* < 0.05) compared with the DAC and LINC00599 Inhibit groups. The ki-67 expression in tumor tissues was detected using immunohistochemistry (Figure 7(c)); the DAC group and LINC00599 Inhibit group showed significantly reduced ki-67 expression compared with the control group (*P* < 0.05); and the DAC group + LINC00599 Inhibit group significantly reduced ki-67 expression compared with the DAC and LINC00599 Inhibit groups (*P* < 0.05).

## 4. Discussion

AML involves malignant cloning of blood stem cells and is frequently associated with multiple genetic alterations [16]. AML is characterized by abnormal proliferation and differentiation of immature myeloid cells and impaired apoptosis, leading to suppression of the hematopoietic system, the complex pathogenesis of which has not been fully elucidated [17]. The discovery of ncRNAs opens new perspectives for the diagnosis, prognosis, and treatment of AML. LncRNAs are involved in the regulation of AML cell proliferation, cell cycle, and apoptosis, and their expression levels can predict the clinical characteristics and outcomes of AML. In recent years, LINC00599 has been shown to be involved in physiological and pathological processes, including carcinogenesis [18], atherosclerosis [19], retinal cone survival [20], and smoking [21]. In this experiment, by constructing LINC00599-interfering cells and treating them with DAC, both alone and together, decreased the proliferation rate of HL60 and CCRF-CEM cells, increased apoptosis and ROS levels, decreased Bcl-2 expression, and increased Bad and cleaved caspase-3 expression. Therapeutic drugs exert selective cytotoxic effects through the activation of apoptosis or programmed cell death [22]. In contrast, expression of many apoptotic proteins was detected in the HL60 and CCRF-CEM cells treated with DAC in this experiment. Bcl-2 and Bax control the cell survival by decreasing and increasing the permeability of the outer mitochondrial membrane, respectively [23, 24]. Apoptosis involves the production of ROS, which are normally produced continuously as oxidative metabolic byproducts and can be scavenged by antioxidants [25]. However, excessive ROS levels lead to cellular oxidative stress, damage to proteins, DNA, and cell membranes, and activation of death receptor-mediated or mitochondrial apoptotic pathways [26]. In the present study, DAC treatment increased the production of ROS in cells, which may be due to DAC-induced apoptosis in HL60 and CCRF-CEM cells. In addition, ROS are thought to activate multiple redox-sensitive signaling cascades by interacting with the Bcl-2 family of proteins as second messengers [27]. The simplest explanation for DAC activity in cancer may be that it reactivates the expression of tumor suppressor genes that have been silenced by aberrant DNA methylation [28].

In this study, we found that the expression of miR-135a-5p in both HL60 and CCRF-CEM cells was significantly upregulated after DAC treatment or inhibition of LINC00599 expression. The mechanisms of miRNA involvement in the pathogenesis of AML include copy number alterations, changes near oncogenomic regions due to somatic translocations, epigenetic changes, aberrant targeting of miRNA promoter regions by transcription factors or oncoprotein alterations, and dysregulation due to miRNA processing [29–31]. In this experiment, miR-135a-5p mimics, miR-135a-5p interference plasmids, wt LINC00599 3′-UTR, and mutant LINC00599 3′-UTR were constructed to verify the regulatory relationship between the miR-135a-5p and LINC00599. The findings revealed that LINC00599 Inhibit combined with miR-135a-5p mimics significantly reduced the proliferation of HL60 and CCRF-CEM cells and increased apoptosis and ROS levels. The competing endogenous RNAs (ceRNA) machinery indicates that various RNAs share common miRNA response elements (MREs), allowing them to compete to bind miRNAs and achieve mutual regulation. Many studies have demonstrated the ubiquity of ceRNA machinery in post-transcriptional regulation, and its role in various diseases, especially tumors, has received widespread attention [32]. The ceRNA mechanism is used in the pathogenesis, diagnosis, treatment, and prognostic prediction of most tumors, including AML. miRNAs can exert powerful regulatory functions in a wide range of areas, and their importance in tumors has been confirmed in numerous studies. This result further confirms that lncRNAs, in combination with miRNAs, play a ceRNA role and thus affect the survival of HL60 and CCRF-CEM cells. In addition, we verified that DAC affects tumor proliferation via LINC00599 by culturing human acute promyelocytic leukemia cells CCRF-CEM and subcutaneously inoculating them into nude mice. Treatment with DAC or the LINC00599 Inhibit alone and in combination reduced the long diameter, short warp, volume, and mass of tumors in nude mice, increased the expression of miR-135a-5p, and decreased the expression of LINC00599 and ki-67. We further verified that DAC regulates the function of miR-135a-5p and its targets by affecting the expression of LINC00599, which in turn affects cell proliferation and apoptosis.

## 5. Conclusion

In summary, this study revealed that DAC inhibited the proliferation of HL60 and CCRF-CEM cells and increased apoptosis and the expression level of miR-135a-5p. By constructing miR-135a-5p mimics, miR-135a-5p interference plasmids, wt LINC00599 3′-UTR, and mutant LINC00599 3′-UTR, we demonstrated that LncRNA in combination with miRNA plays ceRNA function and affects cell survival. We concluded that DAC may regulate the function of miR-135a-5p and its targets by affecting the expression of LINC00599, which in turn affects cell proliferation and apoptosis. This study provides a theoretical reference for DAC to improve the clinical treatment of AML.

## Data Availability

The data used to support the findings of this study have been included in this article.

## References

[1] Hwang D., Kim M., Park H. (2019). Natural products and acute myeloid leukemia: a review highlighting mechanisms of action. *Nutrients*.

[2] Liu Y., Cheng Z., Pang Y. (2019). Role of microRNAs, circRNAs and long noncoding RNAs in acute myeloid leukemia. *Journal of Hematology & Oncology*.

[3] Wang Z., Yang B., Zhang M. (2018). lncRNA epigenetic landscape analysis identifies EPIC1 as an oncogenic lncRNA that interacts with MYC and promotes cell-cycle progression in cancer. *Cancer Cell*.

[4] Zhao H., Zhu H., Huang J. (2018). The synergy of Vitamin C with decitabine activates TET2 in leukemic cells and significantly improves overall survival in elderly patients with acute myeloid leukemia. *Leukemia Research*.

[5] Cany J., Roeven M. W. H., Hoogstad-van Evert J. S. (2018). Decitabine enhances targeting of AML cells by CD34(+) progenitor-derived NK cells in NOD/SCID/IL2Rg(null) mice. *Blood*.

[6] Llave C., Xie Z., Kasschau K. D. (2002). Cleavage of Scarecrow-like mRNA targets directed by a class of arabidopsis miRNA. *Science*.

[7] Jarroux J., Morillon A., Pinskaya M. (2017). History, discovery, and classification of lncRNAs. *Advances in Experimental Medicine and Biology*.

[8] Kopp F., Mendell J. T. (2018). Functional Classification and Experimental Dissection of Long Noncoding RNAs. *Cell*.

[9] Esteller M. (2011). Non-coding RNAs in human disease. *Nature Reviews Genetics*.

[10] Li H., Wang P., Liu J. (2020). Hypermethylation of lncRNA MEG3 impairs chemosensitivity of breast cancer cells. *Journal of Clinical Laboratory Analysis*.

[11] Liu Z., Hua Y., Liu C. (2019). miRNa-494 inhibits apoptosis and promotes autophagy of acute myeloid leukemia cells by downregulating FGFR2. *Minerva Endocrinology*.

[12] Zhao T. T., Liu X. (2019). LncRNA-H19 inhibits apoptosis of acute myeloid leukemia cells via targeting miR-29a-3p. *European Review for Medical and Pharmacological Sciences*.

[13] Meng H., Han L., Hong C. (2018). Aberrant lncRNA expression in multiple myeloma. *Oncology Research*.

[14] Li H., Tian X., Wang P. (2020). LINC01128 resisted acute myeloid leukemia through regulating miR-4260/NR3C2. *Cancer Biology & Therapy*.

[15] Wang Z., Liu J., Yang Q. (2022). LncRNA MIAT upregulates NEGR1 by competing for miR-150-5p as a competitive endogenous RNA in SCIRI rats. *International Journal of Genomics*.

[16] Liu H., Liu M., Zhang J. (2020). Downregulated miR-130a enhances the sensitivity of acute myeloid leukemia cells to adriamycin. *Molecular Medicine Reports*.

[17] Pelcovits A., Niroula R. (2020). Acute myeloid leukemia: a review. *Rhode Island Medical Journal*.

[18] Fu Q., Li S., Zhou Q. (2019). Low LINC00599 expression is a poor prognostic factor in glioma. *Bioscience Reports*.

[19] Shan K., Jiang Q., Wang X. Q. (2016). Role of long non-coding RNA-RNCR3 in atherosclerosis-related vascular dysfunction. *Cell Death and Disease*.

[20] Sanuki R., Onishi A., Koike C. (2011). miR-124a is required for hippocampal axogenesis and retinal cone survival through Lhx2 suppression. *Nature Neuroscience*.

[21] Parker M. M., Chase R. P., Lamb A. (2019). RNA sequencing identifies novel non-coding RNA and exon-specific effects associated with cigarette smoking. *BMC Medical Genomics*.

[22] Pfeffer C. M., Singh A. T. K. (2018). Apoptosis: a target for anticancer therapy. *International Journal of Molecular Sciences*.

[23] Chen Q., Xu H., Xu A. (2015). Inhibition of Bcl-2 sensitizes mitochondrial permeability transition pore (MPTP) opening in ischemia-damaged mitochondria. *PLoS One*.

[24] Edlich F., Banerjee S., Suzuki M. (2011). Bcl-x(L) retrotranslocates Bax from the mitochondria into the cytosol. *Cell*.

[25] Wang X. D., Li C. Y., Jiang M. M. (2016). Induction of apoptosis in human leukemia cells through an intrinsic pathway by cathachunine, a unique alkaloid isolated from Catharanthus roseus. *Phytomedicine*.

[26] Redza-Dutordoir M., Averill-Bates D. A. (2016). Activation of apoptosis signalling pathways by reactive oxygen species. *Biochimica et Biophysica Acta*.

[27] Di W., Khan M., Rasul A. (2014). Isoalantolactone inhibits constitutive NF-*κ*B activation and induces reactive oxygen species-mediated apoptosis in osteosarcoma U2OS cells through mitochondrial dysfunction. *Oncology Reports*.

[28] Herman J. G., Baylin S. B. (2003). Gene silencing in cancer in association with promoter hypermethylation. *The New England Journal of Medicine*.

[29] Agirre X., Martínez-Climent J., Odero M. D. (2012). Epigenetic regulation of miRNA genes in acute leukemia. *Leukemia*.

[30] Zhang H., Kang J., Liu L. (2020). MicroRNA-143 sensitizes acute myeloid leukemia cells to cytarabine via targeting ATG7- and ATG2B-dependent autophagy. *Aging*.

[31] Huang Y. D., Lu Q. Y. (2017). Research Progress on the Drug Resistance Mechanism of Acute Myeloid Leukemia Mediated by the MicroRNA -Review. *Zhongguo Shi Yan Xue Ye Xue Za Zhi*.

[32] Cheng Y., Su Y., Wang S. (2020). Identification of circRNA-lncRNA-miRNA-mRNA competitive endogenous RNA network as novel prognostic markers for acute myeloid leukemia. *Genes (Basel)*.

